# Suicide Prevention Mobile Apps: Descriptive Analysis of Apps from the Most Popular Virtual Stores

**DOI:** 10.2196/13885

**Published:** 2019-08-13

**Authors:** Gema Castillo-Sánchez, Ismael Camargo-Henríquez, Juan Luis Muñoz-Sánchez, Manuel Franco-Martín, Isabel de la Torre-Díez

**Affiliations:** 1 Department of Signal Theory and Communications, and Telematics Engineering University of Valladolid Valladolid Spain; 2 Higher Technical School of Computer Engineers Polytechnic University of Madrid Madrid Spain; 3 Psychiatry Service Rio Hortega University Hospital Valladolid Spain; 4 Psychiatry Service Zamora Hospital Valladolid Spain

**Keywords:** apps, prevention, suicide, virtual store, analysis

## Abstract

**Background:**

Provision of follow-up and care during treatment of people with suicidal intentions is a challenge for health professionals and experts in information and communications technology (ICT). Therefore, health professionals and ICT experts are making efforts to carry out these activities in collaboration by using mobile apps as a technological resource.

**Objective:**

This study aimed to descriptively analyze mobile apps aimed at suicide prevention and to determine relevant factors in their design and development. In addition, it sought to analyze their impact on the support of treatment for patients at risk for suicide.

**Methods:**

We considered 20 apps previously listed in the article “Mobile Apps for Suicide Prevention: Review of Virtual Stores and Literature” (de la Torre et al, JMIR mHealth uHealth 2017;5[10]:e130). To find the apps in this list, the most popular app stores (Android and iOS) were searched using the keyword “suicide prevention.” The research focused on publicly available app information: language, platform, and user ratings. The results obtained were statistically evaluated using 16 parameters that establish various factors that may affect the choice of the user, and the consequent support that the app can offer to a person at risk for suicide.

**Results:**

Of the 20 mobile apps, 4 no longer appeared in the app stores and were therefore excluded. Analysis of the remaining 16 apps sampled showed the following: (1) a high percentage of the apps analyzed in the study (n=13, 82%) are provided in English language; (2) the sampled apps were last updated in 2017, when only 45% of them were updated, but the constant and progressive update of treatments should be reflected in the apps; and (3) the technical quality of these apps cannot be determined on the basis of the distribution of scores, because their popularity indices can be subjective (according to the users). User preference for a particular operating system would require further, more specific research, including study of the differences in the technical and usability aspects between both platforms and the design of medical apps.

**Conclusions:**

Although there are positive approaches to the use of apps for suicide prevention and follow-up, the technical and human aspects are yet to be explored and defined. For example, the design and development of apps that support suicide prevention should be strongly supported by health personnel to humanize these apps, so that the effectiveness of the treatments supported by them can be improved.

## Introduction

The recent significant increase in the use of mobile devices can be highlighted as a high-impact technological revolution. The recent increase in health-oriented mobile technologies and apps (mobile health) developed to construct a new modality for assistance and treatment of patients is evident, and the data on people’s health collected by these apps are valuable [2].

Technically, the primary objective of mobile apps is to offer, and facilitate access to, information and knowledge without restrictions of time or physical space. This possibility of technological ubiquity creates opportunities for new forms of use and communication from these resources [3-5]. Thus, mobile apps become a possible and real alternative for the treatment of mental health problems, especially for people at risk for suicide in follow-up and treatment [6]. As Larsen et al [7] pointed out, “Apps can be especially useful for suicide prevention interventions, because of their ability to deliver support and interventions in situ and at times of crisis.”

In recent years, statistics related to acts and attempts of suicide or self-injury seem to maintain a concerning growing trend. The World Health Organization estimates that more than 800,000 people worldwide die of suicide each year; in addition, suicide is the second leading cause of death among young people aged 15-29 years [8].

Several studies [9,10] have identified the positive effects of new technologies in combating and preventing suicidal behavior. For example, the academic works of Saulsberry et al [1], Carew et al [11], and Hetrick et al [12] indicated different technological resources that can help people in these situations. Thus, discussion and help forums, online treatments, or search for information on the internet has created an interesting positive impact. Similarly, mobile apps have found their niche as technological resources to support this problem.

Some studies on the subject (eg, [13]) suggest that apps that include patient-generated information can be used to optimize outcomes and reduce risks in specific health problems such as suicide. They are also able to identify and understand the technical determinants (eg, design, perceived usefulness, perceived ease of use, and autonomy) that promote the adoption of apps as a habit, which contributes to the patients’ well-being or may lead to the prevention of a mental condition through its previous manifestations (behaviors) or symptoms.

A randomized trial of the effectiveness of the *MoodHacker* app showed interesting results regarding depression symptoms among adult patients who used the app compared with patients who used websites [14]; in the trial with a 6-week follow-up, the app showed significant effects on the symptoms of depression, with η^2^=0.021 among employed adult patients who used the app compared with subjects with the same condition but with access to websites instead of the app. The app also had stronger effects on individuals with access to an employee assistance program (η^2^=0.093). For all users, the *MoodHacker* program showed an improvement in work absence and the mediating factors of behavioral activation, negative thoughts, and knowledge of depression self-care. The study also reported that through this app and the guidance of clinical advisors, the effectiveness of the follow-up for patients who seek support for their treatment, without disconnecting from the human bond, can be significantly improved. Thus, mobile apps that are effectively designed and implemented for mental health treatments should follow particular guidelines to attain the objectives set for the patient.

This study aimed to analyze mobile apps for suicide prevention in order to identify and characterize the main factors, variables, or parameters used for the design and development of these types of mobile apps. In addition, we aimed to determine whether these apps support potential suicide-prone patients in their functionality and thus provide a series of reflections that reinforce previous contributions established by other studies [15,16,17]. This goal may be accomplished by establishment of a simple and clear methodology for identifying the characterizations of these apps and the effect they may have on suicide prevention. This study is an extension of a previous publication [18] and complements it by adding a statistical and descriptive analysis of the characteristics of the 20 previously investigated apps.

## Methods

### Overview

On the basis of the experience of mental health professionals from Hospital de Zamora, Castilla y León (Spain), and information and communications technology (ICT) professionals, the following variables were selected to generally describe the publicly available mobile apps in app stores. These variables or parameters were selected per the study’s objective to quantitatively analyze a set of apps aimed at suicide prevention. The research focused on publicly available app information: language, platform, and user ratings.

These apps were originally listed in a previous publication in 2017 [18] and initially consisted of a list of 20 apps. However, in 2018, the apps were reverified, as four of them no longer appeared in the app stores; this difference was due to the variability of the duration for which the apps are available in the app store. For iOS, apps remain valid for more than 9 months but for Android, they remain valid for a shorter time [17]. The apps *Not Even One*, *Suicide Helplines in India*, *Suicide Thoughts*, and *Suicide or Survive* were excluded, because they did not appear in the app search in 2018. Thus, this section presents the analysis of 16 mobile apps in app stores.

### Selection Criteria

Methodologically, a total of 18 variables were established to characterize the technical and functional properties of the reviewed apps. These variables generally correspond with the metadata that can be obtained from the revised app stores and offer a global statistical perspective on the apps they manage.

**Table 1:** List of the *Features of Interest*.

Variable name	Variable type	Description
Name	Text	Name of the app
Developer	Text	Developer information
Language	Text	Main language *by default*, in which the functional features of the app are offered to the user (interfaces, guides, help)
Total downloads	Number	Total of downloads
Overall rating	Number	Average rating in the store
Total reviews	Number	Total of reviews
Category	Text	App category according to the store
Screenshots	Categorical	Screenshots available from the app
Current version	Number	Current app version
Last update	Date	Latest app update
In-app purchases	Categorical	Possibility to buy things within the app
In-app ads	Categorical	Advertisements within the app
Download size	Number	Application download size (MB)
Developer country	Text	Developer country information
Access	Categorical	Free/premium app
Platform	Categorical	Platform type (Android / iOS)
User journey	Categorical	User guidance on first use
Description	Text	As given by the developer

These variables are listed, defined, and described in Table 1 and are used to define the set of criteria to include, classify, and organize the analyzed apps. This series of properties of the apps selected is called *Features of Interest*.

The variables, or *Features of Interest*, including the name, developer, and total downloads (Table 1) help identify the app and compile a series of descriptive data to distinguish and evaluate them. The variables user journey, screenshots, in-app purchases, and in-app ads allowed us to evaluate characteristics of the apps that could influence their download and use by people in treatment and follow-up.

In addition, the rest of the *Features of Interest* presented here are available in app stores and allow us to determine, for example, the group of people who can use them when estimating the Language variable. This is helpful because these apps can be customized to the language of the population to which suicide attention or prevention can be provided. Thus, after selecting and determining the variables to be used, the data collected are normalized and cleaned (eg, formatting, spellings, duplication, and extra spaces) to provide a consistent basis for analysis. To subsequently construct the final set of data (data that will be used with the analysis tools), we started by using the initial raw data acquired from the search in the app stores in the following process: selecting the data captured in tables, organizing the records, and reviewing the attributes of these data according to the variables.

### Search Strategy, Data Synthesis, and Analysis

**Figure 1:**
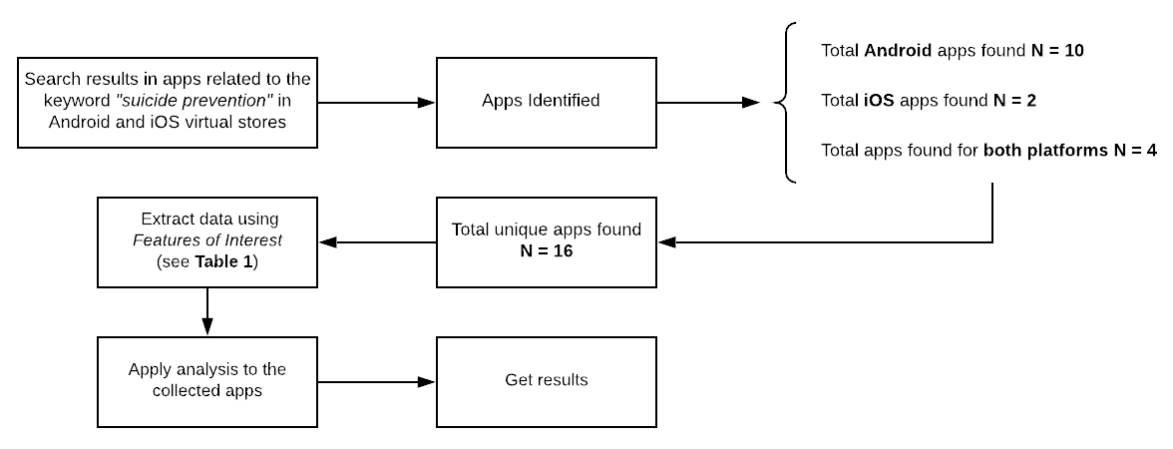
Data search and analysis strategy.

We applied the *Features of Interest* to a data search and analysis strategy proposed in Figure 1. This figure describes the process of data acquisition through app searches based on keywords, classification, and organization of the intermediate results obtained from the virtual stores (Android and iOS); selectivity and analysis of data through the *Features of Interest*; and the results obtained and presented in this paper.

As shown in Figure 1, we searched the app stores using the keyword “suicide prevention” and identified 10 Android apps, 2 iOS apps, and 4 apps for both platforms. The searches were conducted and results were obtained in October 2018, leading to identification of 16 apps presented in this study. The series of parameters described in Table 1 were applied to this set as the data extraction criteria. Our search is similar to those used in previous studies (Android [19], iOS [20], both [18]). In our first study [18], an exploratory and descriptive investigation was carried out, which allowed us to derive basic aspects that are useful for this study, of which the *Features of Interest* was the most important.

As a preliminary result, we obtained a list of apps that matched the search criteria: 10 mobile apps in the Android platform, 2 mobile apps in the iOS platform, and 4 apps found could be run on both platforms. From this list, individual review of each app was performed by evaluating and recording the values that each app contributed as per the previously defined *Features of Interest* (Table 1). The completeness of the recording of these values for each app allowed us to analyze the general characteristics of the apps, with reasoning based on the quantification of the values obtained for each feature of interest. Finally, the results produced were organized and presented statistically.

## Results

### Features of Interest

This section briefly describes the results obtained from the analysis of the 16 selected apps. The general considerations provided in Tables 2 and 3 correspond to the most relevant *Features of Interest* previously established.

**Table 2:** *Features of Interest* of the apps (N=16).

Feature		Values, n (%)
**Language**		
	English	13 (82)
	Dutch	1 (6)
	Spanish	1 (6)
	Multi-language	1 (6)
**Developer country**		
	Canada	1 (6)
	Scotland	1 (6)
	Spain	1 (6)
	United Kingdom	2 (13)
	United States	6 (38)
	Not specified	5 (31)
**Screenshots**		
	Yes	15 (94)
	No	1 (6)
**In-app ads**		
	Yes	1 (6)
	No	15 (94)
**Platform**		
	Android	10 (63)
	iOS	2 (13)
	Both	4 (25)
**User journey**		
	Yes	12 (75)
	No	4 (25)
**Last update**		
	2013	1 (6)
	2015	1 (6)
	2016	4 (25)
	2017	4 (25)
	2018	6 (38)

**Table 3:** App download, rating, and review data (part of the *Features of Interest* list).

App name	Overall rating^a^	Total reviews, n	Total downloads, n
DMHS^b^ Suicide Prevention	3.80	4	100
Suicide Prevention Help Squads	3.70	14	1000
Prevent Suicide - NE Scotland	5.00	27	1000
HELP Prevent Suicide	4.00	5	500
Ask & Prevent Suicide	4.60	13	1000
Suicide? Help!	2.90	72	10000
Prevensuic	4.30	49	1000
Stay Alive	3.80	201	10000
Suicide Preventive	3.20	28	5000
Suicide Lifeguard	3.10	40	1000
Suicide Safety Plan	3.90	179	10000
A Friend Asks	4.20	17	5000
Virtual Hope Box	4.20	611	10000
Operation Reach Out	4.00	24	1000
We Care	5.00	4	1000
R U Suicidal	2.70	30	1000

^a^1=poorly rated, 5=highly rated.

^b^DMHS: Durham Mental Health Services

#### Language

A high percentage of the apps analyzed in the study (82%) are presented in English. The remaining 12% are offered to their users in Spanish or Dutch, and only 6% of the apps are presented with multilingual capability. Notably, the proportion of apps in a particular language (English) could limit the app’s access to patients who do not know that language, reducing the impact and use of these apps for patients with a regional language other than those offered by the apps analyzed.

#### Developer’s Country

The predominance of English language in the analyzed apps was correlated with the countries of origin in which they were developed: 63% of the apps reviewed were developed collectively in the United States (38%), the United Kingdom (13%), Canada (6%), and Scotland (6%). On the contrary, only one of the apps was developed in Spain (6%). Finally, the origin could not be identified for 31% of the apps.

#### Total Downloads

Table 3 lists the most outstanding apps that, at the time of the analysis, had an average of 10,000 downloads. The following four apps were identified in this range of downloads: *Suicide? Help*, *Stay Alive*, *Suicide Safety Plan*, and *Virtual Hope Box*. The following were apps with 5000 downloads: *Suicide Preventive* and *A Friend Asks*. The apps *HELP Prevent Suicide* and *DMHS Suicide Prevention* had the lowest download average numbers of 500 and 100, respectively. Finally, the remaining apps in the evaluated set followed the trend of at least 1000 downloads. A possible indicator that can influence download averages could be the popularity of such apps among users, personal (or clinical) recommendations, and ranking within the app store. However, these are not determinants to qualify the relative effectiveness of the apps as tools for suicide prevention or support treatments. The total number of downloads was obtained from the apps available on the Android platform.

#### Overall Rating

During analysis, we found that use of Overall Rating as a comparative indicator in relation to the total downloads of the apps reinforces the argument that the download index or popularity of an app is not directly related to its effectiveness as a tool to support treatments and suicide prevention. Table 3 shows that apps that obtained a high index of downloads (*Suicide? Help!*, *Stay Alive*, *Suicide Safety Plan,* and *Virtual Hope Box*) now present average ratings of 2.90, 3.80, 3.90, and 4.20 (on a maximum rating of 5.00), respectively. However, they do not exceed the rating of 5.00, similar to apps with lower downloads, such as *Prevent Suicide - NE Scotland* or *We Care*, that had an average of 500 and 1000 downloads, respectively.

#### Total Reviews

Table 3 presents a new comparison variable. The apps most commented on or reviewed by the public (users and relatives) present heterogeneous values, highlighting only three apps with high values: *Stay Alive* (201 reviews), *Suicide Safety Plan* (179 reviews), and *Virtual Hope Box* (611 reviews). The total number of reviews could be associated with the popularity of the apps due to the influence of the total downloads on the rating given to each app. However, the rating could continue to be a subjective metric due to its reliance on opinions with a strong component of feelings (negative or positive) toward the app.

#### Screenshots

Most of the evaluated apps (n=15, 94%) presented screenshots showcasing the app’s features to potential users before they downloaded the app; such screenshots offer a brief overview of the app’s esthetic aspects and most relevant functionalities.

#### Last Update

An aspect valued in the evaluation and analysis of apps is the date of updates or the frequency in which their developers include new features in the apps. For example, many of the apps (n=6) were updated until 2018 (Table 2). However, these values are relative and, to a great extent, depend on the continuity of the app by its developers and their affiliation with private or public organizations that collaborate in the app’s development and growth (through funding, inclusion of new treatments, helplines, and groups).

#### In-App Ads

We observed a trend of noninclusion of ads within the content of the apps. This feature is useful, as it avoids detracting the patient’s concentration or overwhelming the patient with unwanted advertising that would compromise the objectives of the app (treatment, help, and follow-up) and thus discourage app use due to disinterest.

#### Platform

Two platforms (Android and iOS) and their corresponding app stores were evaluated. We observed a marked trend of development of apps oriented toward the Android platform (63%), which can be associated with the popularity and assortment of mobile devices (cell phones and tablets) for this platform. In addition, notably, there was an orientation toward apps with a duality of operation between platforms (Android ↔ iOS). In our analysis, 25% (n=4) of the reviewed apps showed this duality by providing access to users of one or the other platform. Finally, 13% (n=2) of all apps under study run on the iOS platform natively.

#### User Journey

An important feature of this study was consideration of the user journey as a teaching resource within the evaluated apps; 12 apps reviewed (75%) present this resource as a part of their design, with 4 apps not using this component. Designers seem to support the good use of the app by the patient in a guided manner, which promotes their care and supports the objectives of the app itself in relation to the patient.

## Discussion

### Principal Findings

Our findings suggest that *Virtual Hope Box* has many factors that fulfill the investigated parameters. First, it is the only app among the apps analyzed that offered six languages, which allows it to reach a larger audience in different regions. This app had the highest numbers of downloads (n=10,000) and reviews (n=611) from among the 16 apps, possibly due to this reason. In addition, it had an average rating of 4.20 and no in-app advertisements or purchase options, which may be discouraging to the users. Another important and significant aspect is that the app is updated frequently; its last update was in October 2018. Thus, it maintains constant support. Finally, the users’ perception of the app with regard to the omission of the word “suicide” as part of the app's name is notable. The word “suicide” is often associated with negative emotions, and use of positive words such as “hope” as part of the app name is a better option for the prevention of such incidents.

The *Virtual Hope Box* was developed at the National Center for Tele-Health and Technology (US Department of Defense) and is based on the analog version of the hope box concept, a therapeutic tool used by clinicians to help patients with depression or suicidal tendencies to redirect their negative thoughts toward reflecting on reasons for living instead [21]. This app is particularly interesting because, based on the evaluated results, the technical elements of its use and design could be used in the development of similar apps.

### Limitations

That there were some limitations to the set of apps sampled, and these limitations condition the presentation of the analyzed data. First, combining the Android and iOS apps into one row has resulted in a major technical flaw. For example, in case the app is available for both Android and iOS, the data recorded for downloads, ratings, and all other variables do not clearly indicate whether the data are for the Android or iOS platform. Each app’s data should have been recorded separately. Second, as the number of apps (N=16) was very low, we could not prove statistical significance for any of our results. For example, there were only two iOS apps; therefore, no result could be validated. Third, this study did not examine the characteristics associated with the app content, clinical appropriateness, compliance with suicide prevention policy guidelines, or the scientific evidence base for the included apps, which limits the extent to which this analysis can evaluate such apps beyond providing an overview of details such as current usage and user ratings.

### Conclusions

Establishing parameters to identify mobile apps for monitoring, prevention of, and provision of attention to suicide is not a trivial task. However, this joint effort between ICT experts and mental health professionals has made it possible to generally describe the metadata provided in app stores in terms of the apps offered for these purposes. These data allow us to observe certain behaviors of interest related to downloads, determine the predominant language, use technological resources, and confirm previously established characteristics. Analysis of the 20 mobile apps sampled showed the following main findings: (1) a high percentage of the apps analyzed in the study (82%) are provided in English language; (2) the sampled apps were last updated in 2017, when only 45% of them received an update, but the constant and progressive update of treatments should be reflected in the apps; and (3) it is impossible to accurately determine the technical quality of these apps based on the distribution of scores (Table 3) because the popularity indices shown in Table 3 can be subjective (according to the users). Therefore, it would be necessary to obtain information on the design protocols, development, verification of applications, technical resources for the platforms, tools, engineering techniques used, etc. Thus, further evaluation is needed to determine this accurately.

Finally, the apps designed for suicide prevention and follow-up for patients at risk for suicide could be more successful if they were actively supported in their design by qualified mental health professionals, who could provide humanization by following up or treating people who, in these critical situations, need help using an app as a communication resource. Thus, the accompaniment of specialized medical personnel becomes a determining factor in suicide prevention because the components that cause it are complex and as variable as the different circumstances faced by people at risk. Facilitating treatment with this type of technology, with an adequate human approach, could save lives, but it will still be a challenge to implement for health professionals working in combination with ICT experts.

### Future Work

Research will continue in this area and will be taken into account for the design and development of a mobile app with the possibility for it to be tested in patients with a suicidal risk.

## Abbreviations

ICT: information and communications technology
